# Treatment of Constipation

**Published:** 1906-05

**Authors:** Theodore Toepel

**Affiliations:** Atlanta, Ga.


### TREATMENT OF CONSTIPATION.*

BY THEODORE TOEPEL, M.D.

ATLANTA, GA.

I was prompted to write this paper by a discussion which took place at a meeting of the Atlanta Medical Society about a year ago, also on account of the wide experience which I have had in prescribing some simple methods of treatment, and the good results derived therefrom. Before I enter upon my chosen subject,

* Read before the Fulton County Medical Society.

I ask your indulgence to a few general remarks by way of introduction.

Life, according to Herbert Spencer, is characterized by the power of living beings to preserve a mobile equilibrium within their environments, or, as he phrases it, by "the continuous adjustment of internal relations to external relations."

Natural recuperative power has been developed, not through the intaking of substances foreign to the organism, but by physical, chemical, and, finally, psychic reactions of the cells, tissues, organs, systems, and, a factor not to be ignored, of the organism as a whole.

We must recognize that disease and recovery are alike vital processes in which the organism itself is the most active agent, and that neither morbific nor therapeutic influences endow the organism with new attributes or introduce into its operations new powers, but we must keep in mind that disease and recovery are often, if not always, one continuous process. A health preserving and health restoring tendency exists; that is a natural endowment, and not the gift of art, and that it is dependent upon the inherent properties of cells, tissues, organs and the organism. Some of these qualities are constantly manifested, while others are evoked only in reaction to perturbing influences. Salutary reactions, however, may be delayed, deficient, or excessive; and thus art must come to the assistance of nature, and therapeusis finds its reason for being. All successful treatment, nevertheless, depends upon the evocation, stimulation and control of the recuperative reactions, together with the suppression, diminution, or neutralization, of antagonistic reactions likewise occurring automatically as the result of extraneous, morbific influences or of internal failures or disturbances.

I have great faith in the power for good of the right drug, given at the right time in the right dose. Nevertheless, a more restricted use may be made of drugs with less danger of harmdoing by reason of mistake in the election of drug, dose or time, by the physician who familiarizes himself with the powers of the remedial agent falling into the group of natural or physiologic therapeutic means.

But all that exists in nature is natural—a drug equally with sunlight, microbe equally with antitoxin; and, under all the circumstances, the disordered functions of the paralytic, for example, are equally physiologic with the co-ordinated functions of the athlete. Moreover, any intervention by the physician in a case of illness, be it merely to enforce rest, or to regulate diet, or to open the windows of the sick-room, is an exercise of his art.

Hippocrates, the empiric, was the father of scientific medicine; the dogmatists were his opponents, and dogmatism is still the enemy of medical progress. Rational empiricism in medicine consists in the orderly arrangement and analysis of facts observed not only in the laboratory, but also at the bedside; and in making, from the data thus established, inductions to the principles of science, and deductions to the applications of art. In the experience to which rational physicians look must be included the whole history of the human organism, and, indeed, so far as these can be learned, a study of the conditions that have affected the living matter before it was human; for in the actions and reactions of living matter with its environment, from the simplest to the most complex, are to be found the influences that have determined, not alone the physiologic, but also the pathologic, development of man, together with the power of the organism to recover from the disturbance that we term disease.

Speculation upon the origin of living matter is enticing, but not profitable. Given the original living matter with its inherent forces and tendencies, and its development into man, and man's coming into his present physical, mental and moral conditions are the resultants of habit and environment. Under these general heads are to be understood the effects of climate and of weather (including heat and cold, the physical and chemical constitution of the atmosphere, sunlight and other light, electricity, etc.), the use of water, food and methods of feeding, rest, and exercise of function, physical and mental, of which last, not the least important phase is one commonly overlooked—emotion.

These and similar influences having helped to make man what he is, may well be employed to remake him when he departs from the normal.

Hoping that this digression has not been too lengthy, I shall now proceed with the discussion of my subject.

Constipation may result from one or more of the following causes: 1. Insufficient quantity of solid food. 2. Too highly nutritious or concentrated food. 3. Insufficient fluid. 4. Astringent food and drinks. 5. Indigestible food. 6. Lack of digestive fluids. 7. Irregularity in diet. 8. Obstruction from overeating. 9. Lack of peristalsis. 10. Lack of exercise. 11. Certain diseases, such as anemia, neurasthenia and hysteria, chronic effectious affections of the liver, stomach and intestines. 12. Habitual drug-taking. 13. Weakness of the abdominal muscles in obesity or from overdistention in repeated pregnancies. 14. The presence of tumors, physiological or pathological, pressing upon the bowel. 15. Atony of the colon, particularly of the muscles of the sigmoid flexure by which the feces are propelled into the rectum, etc.

*Treatment.*—Before prescribing for constipation, interrogate the patient minutely as to his daily habits of life, such as occupation, hours for meals and for exercise, recreation and sleep, the kinds of food and quantity usually eaten, the amount and kinds of fluids drunk, hour for going to stool, the use of stimulants and tobacco, and presence of mental worry and anxiety.

*Diet.*—The principles of diet in the treatment of constipation must be based upon supplying digestible food, which will excite peristalsis either by its bulk or its physical and chemical properties. Vegetable food in general, as distinguished from nitrogenous diet, furnishes a much larger proportion of waste matter. Herbivorous animals have more abundant evacuations than do carnivorous. The most useful articles for constipation are abundant water, coarse brown or bran bread, oatmeal, butter, fresh green vegetables (lettuce, spinach, rhubarb, etc.), prunes, figs, apples (cooked or raw), peaches, berries, buttermilk, apple cider, honey, English walnuts.

Persons suffering from habitual constipation do well to give up the following articles: Eggs, milk, sweets, pastry, puddings made of simple amylaceous substances, such as rice, sago, etc., fried foods, rich gravies, sauces, curry, strong condiments, pickles, cheese, tea, sour or red wines.

*Hydrotherapy.*—Careful observation has convinced me that the addition of some form of hydrotherapy enhances the value of medical gymnastics and dietetic measures, and often succeeds after the latter alone have failed. The hip bath, as recommended by Fleiner, of two to five minutes, temperature of 50° to 68° F., once or twice daily, and an abdominal compress of 50° F. at bedtime, have given excellent results. A form of abdominal douche or hydrotherapeutic massage, from a one-eighth inch nozzle, slowly played over the region of the colon, either at 45° or 110° F., has often furnished satisfactory results. This form of massage can be continued from five to fifteen minutes, if done slowly, and repeated once a day before the usual time for evacuation. This form is highly recommended by Nothnagel and Hackel.

In constipation occurring in gouty, anemic or otherwise depreciated patients, a thorough intestinal irrigation with salt solution, administered every day, removes ptomains. If followed by the rapid introduction and outflow of eight ounces of water at 40° F., the reflex stimulation is aroused. It is not advisable to use the enormous colonic irrigation, because of the overdistention and consequent enfeeblement of the bowels. In all cases of constipation tonic hydriatic procedures are of as great value as is muscular exercise, which it may often substitute temporarily in feeble patients.

*Olive Oil Injections.*—This is a very satisfactory measure, and is highly recommended by Kussmaul. Inject from three to ten ounces of warm olive oil in the rectum at bedtime. This is retained during the night, and usually results in an evacuation after breakfast on the following day. If the oil is introduced slowly, at a low pressure by the force of gravity, the patient should have no difficulty in retaining it for the night. The following is described by Dr. Herschel as the best apparatus to be used in giving oil injections:

"A glass funnel of a large relative capacity to its height, provided with a loop by which it can be suspended at a convenient height above the patient, is fitted with about 28 inches of rubber tube of ⅜-inch caliber, and terminates in a nozzle. I have found the colon tube of equal efficiency. The outflow of the oil is controlled by a spring clip. The injections can be made by the patient without assistance. The oil is first heated by placing the bottle containing it in a basin of hot water. The funnel is hung on a nail above the bed. The patient lies on his back directly under it with his hips on a pillow, introduces the tube and waits until the funnel is empty. It is best to commence with five or six ounces and to reduce the dose daily until the smallest amount which will produce an action of the bowels is found. In a few days it will probably be found that a few ounces of oil at bedtime will produce a daily evacuation. The effect may then be tried of using the oil on alternating nights, and as the case progresses the action of the oil will extend over a longer period and will be followed by several daily stools. When this period arrives the injection should be ordered to be taken only on the evening of the day on which the action has not taken place.

*Massage.*—The most common and important use of abdominal massage is to relieve chronic intestinal inactivity. The various manipulations are complex, and when rightly performed require the skill and experience of thoroughly trained operators. The general courses of constipation, as regards conditions of the bowel which may be relieved by massage, are the following:

1. Relaxed abdominal muscles, resulting in prolapse of the bowel and other viscera, and constant stasis of the intestinal contents, with resulting dilation of the colon. The dilatation may exist either in the cecum or the sigmoid flexure, or the entire colon may be affected. In consequence of delay to evacuate the bowel the fecal contents often form hard masses, as the result of the excessive absorption of fluid due to their prolonged sojourn in the colon.

2. Deficient production of bile, due to an inactive state of the liver, either as a result of inactivity in the portal circulation or some local functional derangement. This condition, commonly spoken of as "torpidity" or "biliousness," is one in which the liver fails to perform its work effectively in destroying the ptomains which are received in the blood or are formed in the alimentary canal, or to convert into less toxic forms the leucomains, or tissue poisons, normally developed in the system and prepared by the liver for elimination by the kidneys.

3. Deficient activity in the nerve elements supplying the intestines and controlling the reflexes by which peristalsis is maintained, the contents of the bowels moved along the intestine, and the normal diurnal rhythm maintained whereby the residue reaching the lower part of the colon is regularly discharged from the body.

The immediate indications in relation to the removal of these causes, so far as massage is effective to accomplish it, may be enumerated as follows:

1. Increase of glandular activity by an increase of the activity of the blood current in the portal vein, and stimulation of the abdominal sympathetic ganglia, the nerve mechanisms which control the motor, vascular, and secretory functions of the intestines.

2. Increase of peristaltic activity through a stimulation of the nervous reflexes by which this activity is maintained, and by an increased outflow of bile from the liver, the natural laxative by which rhythmical peristaltic activity is promoted.

3. Relief of passive congestion of the portal system, and of the viscera under the influence of this branch of the circulatory system, especially the liver, spleen, stomach and intestines, thus aiding the return of these structures to a normal state and a consequent restoration of their functions.

4. Mechanical dislodgment of the contents of the colon.

5. Development of the abdominal muscles, thereby increasing intra-abdominal tension, which favors expulsion of the intestinal contents.

6. *Replacement of displaced viscera.* The procedures offered by massage, by which these indications are best met, are the following:

1. *To stimulate the nervous reflexes, and hence the peristaltic, glandular and vascular activities, under control of the abdominal sympathetic.*

(1) *Reflex Stroking.*—With the ends of the fingers make very light strokes in a circular or semicircular direction about the umbilicus. Also make vertical strokes along the sides in the mammary line, and parallel with the rectus muscle. Strokes may also be made over the fourth, fifth and sixth ribs at the sides of the chest. The profound reflex effect produced in patients who are very sensitive is evidence of the strong influence of this procedure upon nervous activity.

(2) *Vibration.*—Strong vibration applied to the abdominal contents has been shown to be one of the most powerful means of stimulating the nervous reflexes, circulation, glandular activity and peristalsis, which can be employed for this part of the body. I am now using the electrical vibrator in place of hand vibration, as I was taught.

(3) *Percussion.*—This is unquestionably the most powerful of all the stimulating means which can be applied to the viscera through the abdominal wall. All the different modes of percussion, viz., tapping, spatting, clapping, hacking and beating, may be usefully employed.

2. *To produce mechanical effects by means of which stasis of the intestinal contents may be overcome and accumulated fecal matter dislodged, at the same time that the circulatory and glandular activities are stimulated.*

For this purpose deep kneading is especially to be recommended. This may be accomplished by a number of different procedures: (1) Digital kneading. (2) Kneading with the closed fists. (3) Kneading with the thumbs. (4) Mass kneading. (5) Palm kneading. (6) Rolling. (7) Massage of the gall bladder.

3. *To strengthen the abdominal muscles.*

Massage alone is not sufficient as a means of developing the abdominal muscles, as is the case with all muscular structure. They must be brought into voluntary action by proper gymnastics. Kneading is the most effective procedure afforded by massage for stimulating development of the abdominal muscles. Both superficial kneading and deep kneading may be employed.

*Medical Gymnastics.*—In most cases gymnastics is sufficient alone, and is what the patients generally wish, because it is less troublesome and cheaper. To be effectual, gymnastic movements must be used perseveringly; they should be gone through every day at least three times. It is important to impress upon the patient that the treatment should not stop as soon as improvement begins, but it should be continued even a few months after, if good health is to be gained. Every patient treated this way should be advised to rub his abdomen himself, for a relapse can easily occur, but can also easily be avoided, if the patient sacrifices ten to fifteen minutes daily on an abdominal kneading.

Gymnastic exercises for constipation:

1. Stretch stride standing, bending forward.
2. Stretch stride standing, bending obliquely outward with flexion and extension.
3. Stretch stride standing, bending forward, swing arms down-back (wood-chopper).
4. Sitting trunk extension and flexion.
5. Back lying hip balancing, knee flexion and extension, etc.

*Mechanical Massage.*—Numerous instruments have been devised to imitate the action of the hands, but as yet no device has been found quite to equal the human hand for the administration of kneading movements. One of the most useful of all the several forms of mechanical massage is mechanical vibration. Under this heading may be appropriately mentioned the "cannon-ball massage." A cannon-ball covered with leather is a valuable mechanical accessory in the application of abdominal massage. It should weigh from four to six pounds, and should be rolled upon the abdomen, following the course of the colon from right to left. It should be used for fifteen minutes morning and evening, on an empty stomach.

Dr. H. Beer (Klinisch-therapeutische Wochenschrift), describes a mechanical laxative for infants. He uses the bulbous end of an ordinary rectal thermometer covered with vaseline, and inserted per rectum, as in taking the temperature. It is then gently pressed against the sphincter ani and, keeping it in the axis of the rectum, it is made to describe smaller and larger circles, as in dilating the sphincter. After two or three turns the sphincter is felt to relax, the rectum contracts, the anus opens and the act of defecation takes place reflexly. The effect is very prompt when the rectum is filled, and is absolutely harmless to the child.

In conclusion, I wish to say that I have not touched upon all the physiological remedies, but suffice to say that if the physician studies the remedies mentioned, and the patient, over-drugging would soon be a thing of the past. One thing should not be forgotten in treating obstinate cases of constipation, namely, to insist on regular habits, particularly in the young; whether the desire is present to have an evacuation or not, regularity of habit must be insisted upon.

DISCUSSION OF DR. TOEPEL'S AND DR. AMSTER'S PAPERS.

*Dr. Boynton:* Mr. President, I consider myself fortunate to have been present this evening to hear three such excellent papers.

I think, perhaps, if we combine the methods advised in the three papers we would have it about right.

The special exercises and massage, as advised by Dr. Toepel, do good in many cases, while the special dieting and massage, as advocated by Dr. Amster, accomplish the result in other cases, and in still other cases, after trying exercise, dieting and massage, we are compelled to fall back on the drugs, as advised by Dr. Baird.

This simply means that each has its field of usefulness.

I would like to ask Dr. Toepel whether during the early months of pregnancy, when constipation is so troublesome, these special exercises or massage would have any harmful effects.

*Dr. Theodore Toepel* (closing): Mr. President and gentlemen,

I first wish to answer Dr. Boynton's question: "Is it advisable to give exercise and massage to a woman who is pregnant and suffers with constipation?" Carefully directed exercises, such as lying on the back and raising and lowering the legs alternately are beneficial, with no danger of a miscarriage. The same is true of massage. It is even better than exercise. Besides relieving constipation, it gives tone to the abdominal muscles and viscera, and consequently to the fetus.

As to the points brought out in the discussion, I wish to say that an up-to-date physician should be acquainted with all the different methods of treatment, drugs as well as physiological measures. He should always find the happy medium and should never become an extremist, having the confirmed idea that there is but one cure for all. Physiological therapeutics have been neglected by physicians and a certain class of people, not always ethical, have taken advantage of this neglect and are now harvesting gains by their unscientific application of physiological measures. Just as little as you can administer the same drug and dose to all your patients, so must you select your cases for different methods of physiological treatments. These natural measures are not only prophylactic, but are just as curative as drugs. This is especially true in cases of constipation, where trunk exercises properly executed have an excellent curative effect.

A *mother* who has a sick child, calling in a physician to advise her, will do all in her power to restore her loved one to health. She will administer the prescribed medicine at the proper time, and will also massage and exercise the little baby, if so instructed by the physician. I can not understand how a true mother can act contrary to the instruction of the physician, if the health or even life of her baby depended upon the execution of his orders.

As it is with the infant, so, too, with the child at school. Where sedentary habits begin, it is the duty of the parents to see that instructions regarding their children's health be properly and regularly executed. Just at this age do physicians sin so much toward a healthy development of their young patients. It is so much easier to say: "Take one pill at bedtime, and come to me again when the box is empty," than to inquire into the habits and environments of the patient and give some common sense directions about diet, exercise, fresh air and bathing. It is the physicians duty to tell his patient of the resources which nature has placed at his command. Young men, invariably habitual drug-takers, to whom I have prescribed exercises and diet, come to me after two or three weeks and tell me that they were relieved from constipation without any drug; but here I always find it necessary to insist that exercise and diet be continued, if no relapse is to take place, because exercise is as necessary for the well-being of the organism as is sleep and food. Often drummers come to me, telling me that they are tired of taking pills, and wish to know a series of exercises which they can take while on the road. I personally know a number of men who carry dumb-bells along in their trunks, and feel better for the little inconvenience that a few vigorously executed exercises in the morning may give them. Only lazy people are fond of inactivity, and for those pills are convenient. Clerks and bookkeepers in banks, office and stores are the most faithful attendants at the gymnasiums. The large department stores in New York, Boston, Philadelphia, Chicago, and in other large cities, have provided gymnasiums for the use of their employees. Here they are allowed to spend an hour during the day to take health-giving exercise, and are thus better able to endure the mental and physical strain which their positions demand of them. The proprietors find this to be a good investment.

As to the regularity with which some people carry out the instructions of their physicians, I think it will balance up pretty well. They will miss to take the pills just as many times as they will fail to take their prescribed exercise and massage in the morning, especially if they should do like the patient who willed a box securely locked to the physician who had attended him in his last illness, and had also been faithful in prescribing for him during many years of his life. With the box were the instructions that only after his death was it to be opened. The doctor on receiving the gift was greatly pleased that he had been remembered by his friend and patient so generously, but great was his chagrin, upon opening the box, to find all the medicine that he had prescribed to his patient during his life, but which he had failed to take. An accompanying note stated that the doctor should remember him kindly to his friends.

Of the two habits, I believe that to take exercise regularly, to eat in moderation and to live otherwise hygienically, is better than to fill up on drugs.

---

### CERIUM OXALATE IN VOMITING.

Within the last few months Sweetnam *(Dublin Journal of Medical Science, February)* has been using cerium oxalate largely in cases of vomiting due directly to gastric disease, usually giving six-grain doses three times a day in adult patients, combined with ten-grain doses of carbonate of bismuth. In almost every case the result has been most satisfactory, and in several instances, when Bismuth alone has failed to cause any marked improvement, a rapid change for the better has appeared when cerium oxalate has been added to the medicine. In gastric ulcer the pain is relieved and the vomiting ceases almost immediately; the same result occurs in chronic catarrhal gastritis, especially the form which is so common among badly nourished young women, whose work deprives them of their proper quantity of fresh air. In cancer of the stomach, although a permanent cure can not be expected, the symptoms are relieved and the patient's life is prolonged and made far more comfortable than by any other means; the pain is lessened, hematemesis ceases and the patients who have been unable to retain the smallest quantity of food taken by the mouth are soon able to retain several pints of milk per diem and also small quantities of the prepared farinaceous foods. This, of course, does not hold good in cases in which the pyloric region is largely involved and in which the symptoms are mainly due to a mechanical obstruction of that opening.

